# Fabrication of ZnO/ZnAl_2_O_4_/Au Nanoarrays through DC Electrodeposition Utilizing Nanoporous Anodic Alumina Membranes for Environmental Application

**DOI:** 10.3390/nano13192667

**Published:** 2023-09-28

**Authors:** Mohamed Shaban

**Affiliations:** Department of Physics, Faculty of Science, Islamic University of Madinah, Madinah 42351, Saudi Arabia; mssfadel@aucegypt.edu

**Keywords:** nanoporous alumina membranes, ZnO, dye removal, pH sensor, nanoarrays, methylene blue and methyl orange

## Abstract

In this study, anodic aluminum oxide membranes (AAOMs) and Au-coated AAOMs (AAOM/Au) with pore diameters of 55 nm and inter-pore spacing of 100 nm are used to develop ZnO/AAOM and ZnO/ZnAl_2_O_4_/Au nanoarrays of different morphologies. The effects of the electrodeposition current, time, barrier layer, and Au coating on the morphology of the resultant nanostructures were investigated using field emission scanning electron microscopy. Energy dispersive X-ray and X-ray diffraction were used to analyze the structural parameters and elemental composition of the ZnO/ZnAl_2_O_4_/Au nanoarray, and the Kirkendall effect was confirmed. The developed ZnO/ZnAl_2_O_4_/Au electrode was applied to remove organic dyes from aqueous solutions, including methylene blue (MB) and methyl orange (MO). Using a 3 cm^2^ ZnO/ZnAl_2_O_4_/Au sample, the 100% dye removal for 20 ppm MB and MO dyes at pH 7 and 25 °C was achieved after approximately 50 and 180 min, respectively. According to the kinetics analysis, the pseudo-second-order model controls the dye adsorption onto the sample surface. AAOM/Au and ZnO/ZnAl_2_O_4_/Au nanoarrays are also used as pH sensor electrodes. The sensing capability of AAOM/Au showed Nernstian behavior with a sensitivity of 65.1 mV/pH (R^2^ = 0.99) in a wide pH range of 2–9 and a detection limit of pH 12.6, whereas the ZnO/ZnAl_2_O_4_/Au electrode showed a slope of 40.1 ± 1.6 mV/pH (R^2^ = 0.996) in a pH range of 2–6. The electrode’s behavior was more consistent with non-Nernstian behavior over the whole pH range under investigation. The sensitivity equation was given by V(mV) = 482.6 + 372.6 e^−0.2095 pH^ at 25 °C with R^2^ = 1.0, which could be explained in terms of changes in the surface charge during protonation and deprotonation.

## 1. Introduction

Due to its unique electrical, catalytic, electronic, and optical capabilities, zinc oxide (ZnO) nanoarrays and films have tremendous potential in a variety of applications, including piezoelectric devices [1], UV light emitters [2], solar cells [3], and photocatalysts [4]. Additionally, gas and electrochemical sensors [5,6], as well as antimicrobial agents [7,8], are designed using ZnO nanostructures. ZnO nanostructures with various morphologies, including nanowires, nanorods, nanotubes, nanoparticles, and nanorings, have been generated effectively using a variety of methods including laser evaporation [9], vapor transport [10], spin coating [11], SILAR [12], spray pyrolysis [12], chemical vapor deposition [13], and electrochemical deposition [14]. However, electrochemical deposition is an efficient and controllable method for the fabrication of ZnO nanoarrays via template-based growth [15,16]. So, the design of ordered porous structures is of interest to scientists and researchers because they possess so many distinctive characteristics [17,18,19]. Surface porosity and order enhance the interaction of the active sites with the electrolyte [20,21]. To implement the majority of the anticipated uses, it has been even more difficult to create nanostructures with appropriate alignment and a uniform size using the conventional lithographic process [22]. One type of ordered porous structure is anodic aluminum oxide membranes (AAOMs), which are very porous and have predetermined shapes and sizes [23,24]. They may be altered to have controlled pore density and periodicity as well as to produce free-standing membranes [25,26]. Additionally, AAOM has high thermal stability as well as simplicity and controllability regarding its pore structure, which may be easily adjusted by optimizing the anodizing components [27]. These unique features, along with the AAOM’s low cost and high throughput, make it the ideal template to produce ZnO nanoarrays. Using AAOM, several ordered ZnO nanostructures have been produced, including nanoparticles, nanotubes, nanowires, and nanorods [28,29]. Yue et al. used the sol-gel technique to develop a ZnO nanotube within the AAOM pores [28]. Zhao et al. reported the electrodeposition-induced agglomeration of 100 nm ZnO nanoparticles in the upper portions of the AAOM channels [29]. This demonstrates that due to the presence of a barrier layer at the bottom of the AAOM pores, prior attempts to build ZnO nanoarrays have only been successful on the upper portions of the channels.

As a result, challenges in the electrodeposition of ZnO nanoarrays inside AAOMs have included obtaining uniformity in the height and diameter of the nanorods, obtaining uniform alignment without distortion, and reaching a high filling factor. Since these issues are related to the presence of alumina barriers or the lack of opening of channels at the bottom of AAOM, it is imperative to completely remove the barrier layers from the bottom of the pores before the electrodeposition. According to Shaban et al. [30], the barrier layer separating the AAOM from the Al substrate can be completely removed by combining cathodic polarization and pore-widening approaches. To determine the effects of removing the barrier layer on the morphology of the produced ZnO nanoarrays, it is important to compare the morphologies of the electrodeposits into AAOMs with and without barrier layers.

From the standpoint of applications, the designed ZnO nanoarrays are anticipated to perform better in various environmental applications such as chemical sensors, catalysts, and adsorbents due to their expected high surface area, small sizes, uniform order, and quasi-continuous controllability of lengths and diameters. This will make it more feasible to use these arrays in electrodevices and chemical sensors to explore their performance. ZnO nanowires’ n-type semiconducting behavior was reported by Xu et al. due to native defects such as oxygen vacancies and zinc interstitials [31]. Also, Chang et al. claim that single crystalline ZnO nanostructures have superior electrical characteristics versus polycrystalline thin films [32]. According to Park et al. [33], the ZnO nanostructure-based device’s high electron mobility of 1000 cm^2^/V.s allows it to run more quickly than its thin-film equivalent. So, the electrical characteristics of nanowires can be modified by varying the carrier concentration and mobility. According to Xu et al., well-aligned ZnO nanorods are crucial for dye-sensitized solar cells (DSSCs), whose electrical mobility is roughly 2–3 orders of magnitude better than that of a TiO_2_ nanoparticle layer [34].

The Kirkendall effect, a mutual diffusion process over a two-metal interface, was effectively used in the solid-state reaction [35]. Yin et al. reported the Kirkendall effect for nanostructures for the first time [36]. The Kirkendall experiment demonstrated two important ideas: atomic diffusion occurs through vacancies, and each metal diffuses at a different mobility. Yang et al. studied the influence of temperature on the coaxial formation of ZnO/Al_2_O_3_ 1D heterostructures [37]. Based on this study, it was necessary to optimize the ratio of pore radius to approximately half the inter-pore separation in order to construct ZnAl_2_O_4_ nanoarrays. Based on this, only a small amount of research has been conducted into the Kirkendall effect of ZnO/Al_2_O_3_ nanostructures. Also, it was found that the addition of a plasmonic ultrathin layer can enhance the performance of a variety of semiconductor materials [4,38,39,40]. All these factors serve as the driving force behind our research on the creation and characterization of highly ordered ZnO nanotubes and ZnO/ZnAl_2_O_4_ nanorod arrays with high filling factors utilizing AAOMs and Au/AAOMs with and without bottom layers for environmental applications. Also, it is important to emphasize the Kirkendall effect in the production of ZnO/ZnAl_2_O_4_ nanoarrays. In this study, AAOMs and AAOM/Au with pore diameters of 55 nm and inter-pore spacing of 100 nm are used to develop ZnO/AAOM and ZnO/ZnAl_2_O_4_/Au nanoarrays with different nanomorphologies using electrodeposition followed by an annealing process. The effect of the electrodeposition current, electrodeposition time, the barrier layer, and the Au coating on the morphology of the resultant nanostructures were explored. Also, the structures and elemental composition of the produced ZnO/ZnAl_2_O_4_/Au nanoarray, for the first time, using the Kirkendall effect was confirmed through EDX and XRD analysis. The designed ZnO/ZnAl_2_O_4_/Au electrode was applied for the removal of organic dyes such as methylene blue (MB) and methyl orange (MO) from aqueous solutions. The kinetics of the adsorption reaction was studied. Moreover, AAOM/Au and ZnO/ZnAl_2_O_4_/Au nanoarrays are also used as pH sensor electrodes. Their sensing capabilities are addressed in terms of their sensor sensitivity, pH range, and detection limit.

## 2. Materials and Methods

### 2.1. Preparation of AAOM and AAOM/Au

To prepare the AAOM, a two-step anodization procedure was performed [30]. Acetone and alcohol were used to degrease high-purity aluminum foils (99.999%), which were then cleaned in distilled water before being electropolished. Under a continuous DC voltage of 50 V at 0 °C, the anodization was performed in a solution of 0.3 M oxalic acid. A solution of phosphoric acid (6 wt%) and chromic acid (2 wt%) was used to dissolve the alumina (Al_2_O_3_) layer that was created during the anodization step at 60 °C. The second anodization was conducted under the same conditions for 5 min to grow high-ordered AAOM. Two sets of AAOMs were produced. For the first set, chemical etching in a 6% H_3_PO_4_ solution for 90 min expanded the pores and weakened the barrier layer. The barrier layer was fully eliminated from the bottom of the AAOM in the second set by combining 10 min of cathodic polarization with 70 min of pore widening.

Au was deposited at pressures of 2 Torr (low vacuum), currents of 13 mA, and distances of 8 cm in front of the Au target using a very basic sputter coating process. For two minutes, Au was coated on a series of AAOMs with the same pore diameter.

### 2.2. Deposition of ZnO Nanostructure within the AAOM

The following procedure was used to electrodeposit ZnO within the AAOM. A layer of Au was sputtered onto one side of the membrane, and Au/AAOM functioned as the working electrode. A volume of 50 mL of 0.01 M Zn(NO_3_)_2_·6H_2_O was combined with 0.31 mL of 0.1 M HNO_3_ to create an electrolyte. During the electrodeposition procedure, various electrodeposition currents were used for varying lengths of time (up to 90 min) at a constant potential difference of 3.2 V and 85 °C. The samples were promptly rinsed with deionized water after deposition. The Zn/Au/AAOM underwent a 24 h oxidation process at 300 °C in the oxygen.

### 2.3. Sample Characterization

To identify the crystallographic characteristics of the produced structures, X-ray diffraction (XRD, X’PertPro MRD, Philips, Westborough, MA, USA) was utilized with Cu_Kα_ radiation (*λ* = 1.5418 Å) and a step of 0.021°. Energy-dispersive X-ray (EDX; Oxford Link ISIS 300 EDX, Concord, MA, USA) analysis was used to study the chemical composition of the samples. Field-emission scanning electron microscopy (FE-SEM, model: ZEISS SUPRA 55 VP and ZEISS LEO, Gemini Column, Troy, NY, USA) was used to conduct morphological analyses on the created nanostructures.

### 2.4. Dye Removal and pH Sensing Applications

To study the dye removal percentage (removal%) of 50 mL methylene blue (MB) and methyl orange (MO) of a concentration of 20 ppm, a 3 cm^2^ ZnO/ZnAl_2_O_4_/Au sample was used. The adsorption tests were carried out with the template still on without etching the Al substrate. The effect of contact time on the dye removal % was conducted up to 55 min and 180 min for MB and MO, respectively, at 25 °C and pH 7. All adsorption tests were conducted on a batch-mode scale. The UV/Vis spectrophotometer (PerkinElmer, Lambda 950, Boston, MA, USA) was used to determine the variance in dye concentration by tracing the absorption peaks at 668 nm and 460 nm for MB and MO, respectively.

To test the photocatalytic reduction properties, a ZnO/ZnAl_2_O_4_/Au photoelectrode was applied for the reduction of MB and MO. The experiments were conducted under an artificial white-light irradiation from a blended metal halide lamp of 400 W at room temperature (25 °C) and pH 7. The measurements of dye concentrations were carried out up to 40 min illumination at 668 nm and 460 nm for 20 ppm MB and MO solutions of a 50 mL volume, respectively, using the UV/Vis spectrophotometer. For the control, the test was carried out under the same conditions for MB under white-light illumination without the existence of the photoelectrode.

The pH sensors utilized in this work, Au/AAOM and ZnO/ZnAl_2_O_4_/Au, were based on straightforward potentiometric measurements for buffer solutions, with pH values ranging from 2 to 9. A multimeter (UT71A, Fluke Corporation, Everett, WA, USA) was used to measure the potential between the working sensor electrode and the saturated calomel electrode (the reference electrode) in a straightforward potentiometric manner.

## 3. Results and Discussion

### 3.1. Morphology and Chemical Composition of AAOM and Au/AAOM

**Figure 1:**
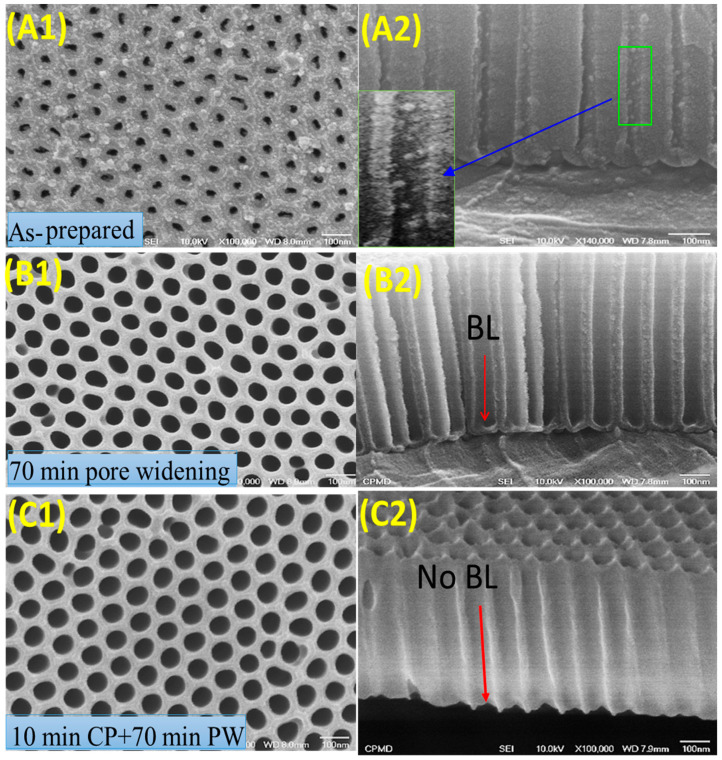
Top and cross-sectional views of AAOMs anodized for 5 min (**A1**,**A2**) as-prepared, (**B1**,**B2**) after 70 min pore widening (PW), and (**C1**,**C2**) after 10 min cathodic polarization (CP) followed by the 70 min pore-widening process. BL refers to the barrier layer. (Scale bare = 100 nm for all images).

FE-SEM images were used to explore the AAOMs’ surface morphologies. Figure 1 depicts the top view and cross-sectional view of an AAOM anodized for 5 min in the following conditions: (A1, A2) as prepared; (B1, B2) after pore widening (PW) for 70 min; and (C1, C2) after cathodic polarization (CP) for 10 min followed by pore widening for 70 min. Due to the presence of certain Al_2_O_3_ flocks inside the nanopores, as seen in the enlarged part of Figure 1(A2), the pores in the as-prepared AAOM exhibited extremely poor regularity. The barrier layer (BL) thickness ranged from 29 to 45 nm, the pore height was 700 nm, the pore diameter was 30 nm, the interpore distance was 100 nm, and the pore density was 1.2 × 10^10^ pore/cm^2^.

It is evident that the flocks inside the pores vanished after 70 min of pore widening, and the surfaces of the pore walls were extremely smooth. Because of the etching caused by the OH^−^ ions that were continuously produced at the bottom of every pore and driven toward the anode to target the bottom barrier layer at a proper cathodic voltage, the thickness of the barrier layer was reduced [41]. Thus, the chemical attack of the OH^−^ ions during this process may result in the dissolution of the barrier layer’s constituent, alumina [30]. Hence, the pore diameter rose to 60 nm and the barrier layer thickness dropped to a range of 17 to 26 nm. That is, the barrier layer was not entirely removed, as shown in Figure 1(B2). With the help of 10 min of cathodic polarization followed by 70 min of pore widening, the barrier layer was completely removed, and the pores widened to 72 nm, as shown in Figure 1(C1,C2). This AAOM can, therefore, be effectively employed to create nanoarrays.

**Figure 2:**
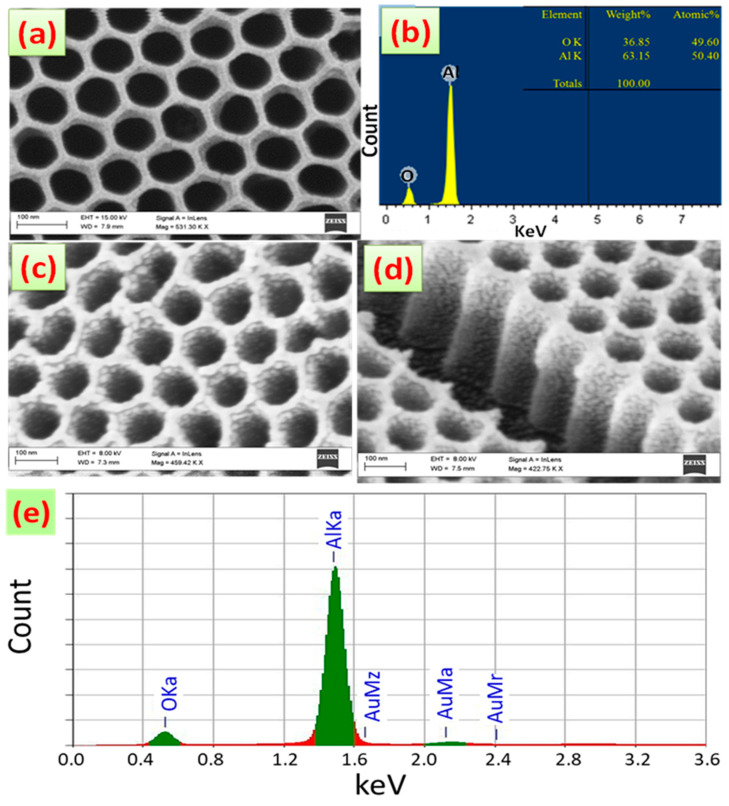
(**a**) Top-view SEM image and (**b**) EDX spectrum of AAOM after 80 min pore-widening process combined with 10 min cathodic polarization; (**c**) top-view and (**d**) cross-sectional-view SEM images and (**e**) EDX spectrum of AAOM sputtered with Au for 2 min. (Scale bare = 100 nm for SEM images).

Figure 2a is a typical top-view SEM image of an AAOM with a 90 nm pore diameter that was produced with the aid of cathodic polarization. Figure 2b displays the EDX spectrum for AAOM to hint at the membrane’s elemental composition. This chart demonstrates the extraordinary purity of the Al_2_O_3_ membrane by revealing that the EDX pattern contained no signs of C, Cr, or S. According to the appended table, the quantitative analysis of Al_2_O_3_ indicates 63.15% Al and 36.85% O. The remaining portion of the Al signal is provided by the Al substrate. Top-view and oblique-view FE-SEM images of AAOM coated with Au for two minutes at 20 W are shown in Figure 2c,d. The top surface of the membrane, the pore walls, and the bottom of the pores were all covered with extremely small Au nanoparticles (<15 nm). The size distribution of these nanoparticles was narrow, and the density of the particles covering the pore walls was quite high and gradually decreased from the top to the bottom of the pores. This Au layer’s average thickness was calculated to be 10 nm. The EDX spectrum of Au/AAOM is shown in Figure 2e. It displays solely Al, Au, and O signals and attests to the designed sample’s high degree of purity.

### 3.2. ZnO-Nanoarray Morphology and Composition

**Figure 3:**
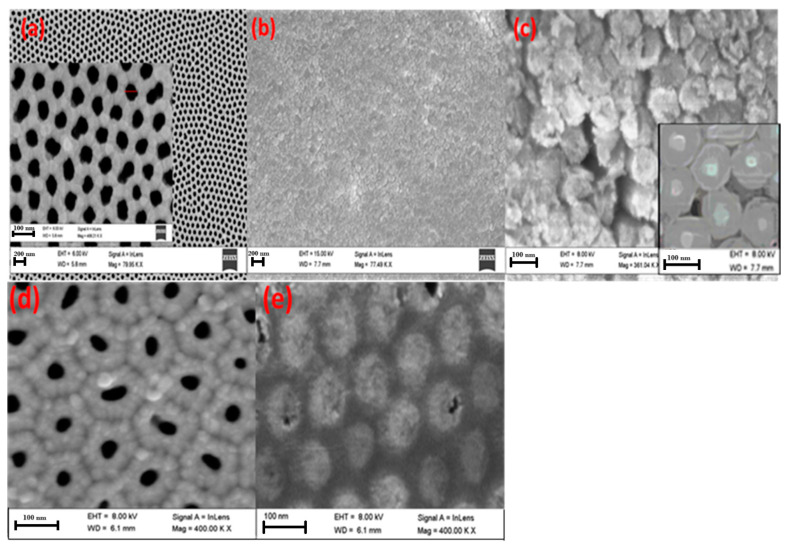
Top-view SEM images of ZnO nanoarrays electrodeposited on 10 nm Au-decorated AAOM without a bottom barrier layer at V = 3.2 V (**a**) I = 0.013 A, t = 20 min and (**b**,**c**) I = 0.050 A, t = 90 min at different magnifications. The inset image of (**c**) confirms the formation of the core–shell morphology of ZnO/ZnAL_2_O_4_. The SEM images (**d**,**e**) show the back side of the formed nanotubes and nanorods after chemical etching of the Al substrate using Al etchant type D at 30 °C.

Figure 3a–c illustrates top-view SEM images of ZnO (a) nanotubes and (b, c) nanorods grown inside the pores of 10 nm Au-decorated AAOMs without bottom barrier layers. The images show that the nanostructures were parallel to each other and vertically oriented to form nanoarrays. The outer diameter of the nanotubes was about 70–80 nm, corresponding to the channels in the AAOM. The diameter of nanorods was around 98 nm, which was very close to the interpore distance. This indicates the diffusion of Zn atoms to the alumina, according to the Kirkendall effect, to form a shell of ZnAl_2_O_4_ around each ZnO nanorod. The inset image of Figure 3c confirms the formation of the core–shell morphology of ZnO/ZnAl_2_O_4_. Moreover, it was found that practically all of the AAOM’s pores were filled and that 100% filling for both nanotubes and nanorods was achieved. The Al substrate was etched, and the SEM images of the back side are displayed in Figure 3d,e for the nanotubes and nanorods to demonstrate that this filling ratio was achieved not just on the top surface. I.e., these images can confirm the growth of nanotubes and nanorods along the entire pores of the AAOM.

**Figure 4:**
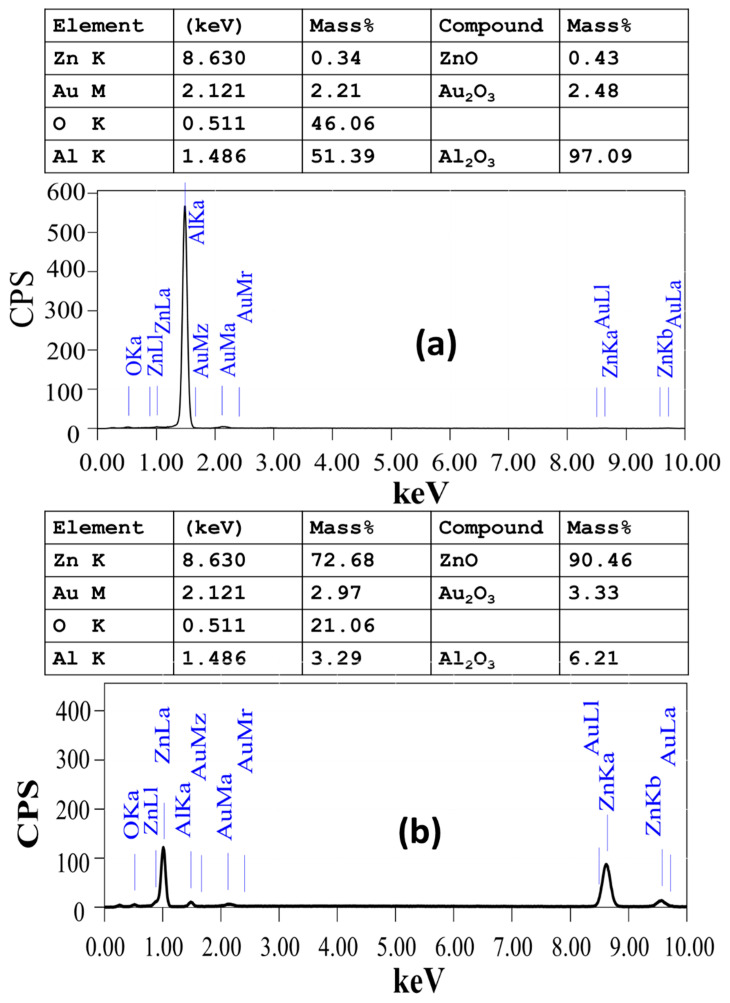
EDX spectra of ZnO nanoarrays electrodeposited on 10 nm Au-decorated AAOMs without bottom barrier layer at V = 3.2 V (**a**) I = 0.013 A, t = 20 min, and (**b**) I = 0.050 A, t = 90 min.

Figure 4 shows the EDX spectra of ZnO-coated Au/AAOM samples at (a) I = 0.013 A, t = 20 min and (b) I = 0.050 A, t = 90 min. The attached tables provide quantitative analyses of the tested samples. The EDX spectra in Figure 4a,b display signals for Al, Au, Zn, and O. This figure shows that no residues of C, Cr, or S from the fabrication solutions were found in the EDX pattern, demonstrating the excellent purity of the deposited ZnO/Au/AAOM nanostructures. The thickness of the deposited Zn on the AAOM significantly rose, as indicated by the increase in the mass ratio of Zn from 0.23% to 72.68%. According to compound analysis, the ratio of ZnO increased from 0.43% to 90.46%. The existence of the Al signal in Figure 4b indicates that the height of ZnO nanorods was less than the interaction volume of electrons with the surface of the sample (~1 μm).

**Figure 5:**
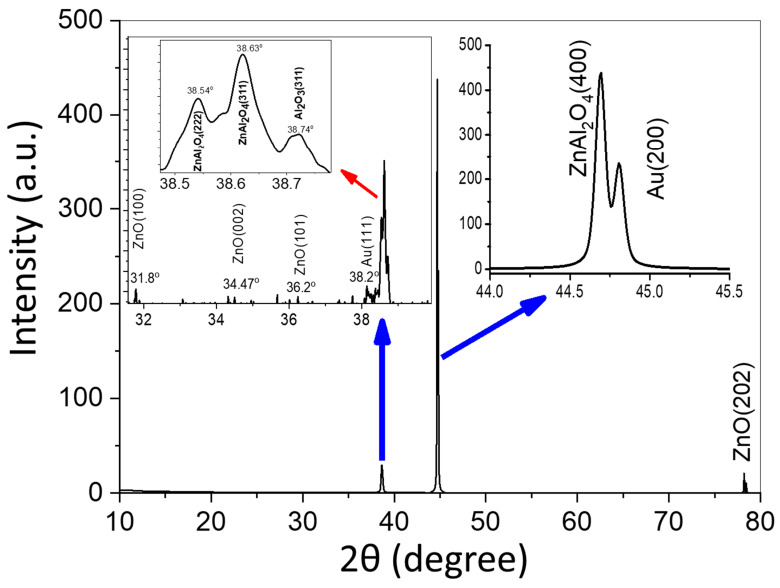
XRD pattern of Au/ZnO/ZnAl_2_O_4_ core–shell nanorod array. The inset is provided to show the existence of very weak ZnO signals in the 2θ range from 31.5° to 40° [JCPDS no. 05-0664].

As shown in Figure 5, the XRD investigation was performed after the annealing at 300 °C. The results indicate reflections corresponding to the crystal planes (222), (311), and (400) for the ZnAl_2_O_4_; (111) and (200) for the Au; and (100) and (202) for the ZnO nanostructures. The intense and sharp diffraction peaks of ZnAl_2_O_4_ indicate that well-crystallized nanorods can be obtained under our synthetic conditions. The data were matched with card no. 04-014-7722 for ZnAl_2_O_4_. Data also showed that the produced nanorods favored aligning themselves along the (400) direction. These results confirm the oxidation of Zn nanorods to ZnO nanorods and the diffusion of ZnO and Al_2_O_3_ to form ZnAl_2_O_4_ nanorods. The reaction may be written as

(1) $$2\;Zn\left(I\right)+O_{2}\left(g\right)\to 2\;ZnO\;\left(s\right)$$

A solid-state reaction now begins which leads to the formation of ZnAl_2_O_4_. This can be described as

(2) $$ZnO+Al_{2}O_{3}\to ZnAl_{2}O_{4}(s)$$

As a consequence of the Kirkendall effect, the diffusion rate of ZnO in the reaction (2) is higher than that of Al_2_O_3_, a behavior that can lead to the formation of a Al_2_O_3_/ZnAl_2_O_4_ nanotube array or ZnO/ZnAl_2_O_4_ nanorod array depending on the relative quantities of ZnO and Al_2_O_3_ [36,37]. It seems from the results that a core of pure ZnO still existed and a Au/ZnO/ZnAl_2_O_4_ core–shell nanorod array was formed. The EDX point analysis of the nanorod is provided as supplementary data (Figure S1) to confirm this core–shell composition.

The Scherrer equation, $CS=\frac{0.94\;\lambda }{\beta \cos\theta }$, was used to obtain the crystallite size (CS) from the XRD chart, wherein the full width at half maximum (FWHM), Bragg’s angle in radians, and X-ray wavelength (CuK_α_ = 0.15418 nm) are presented by β, θ, and λ, respectively [42,43]. Williamson and Smallman’s relation, δ =$\;\frac{N}{CS^{2}}$, was also used to calculate the dislocation density (δ), where N = 1 denotes the minimal dislocation density.

Table 1 includes the calculated values for the crystallite size (CS), d-spacing, and dislocation density, together with the values for FWHM and the relative intensity of the observed peaks in the XRD chart. The values of d-spacing for ZnAl_2_O_4_ in the (311) and (400) planes were 2.33 and 2.03 Å, respectively. The value of the crystallite size for ZnAl_2_O_4_ was lower than the value of the crystallite size for ZnO. The value of the dislocation density varied from 1.81 × 10^−4^ nm^−2^ for ZnO to 2.58 × 10^−4^ nm^−2^ for ZnAl_2_O_4_(400), with Au (200) reporting the lowest value. According to the XRD analysis, the grown ZnAl_2_O_4_ exhibited a high degree of crystallinity along the (400) plane [44].

### 3.3. ZnO Nanorods on the Top Surface of Au/AAOM and AAOM

**Figure 6:**
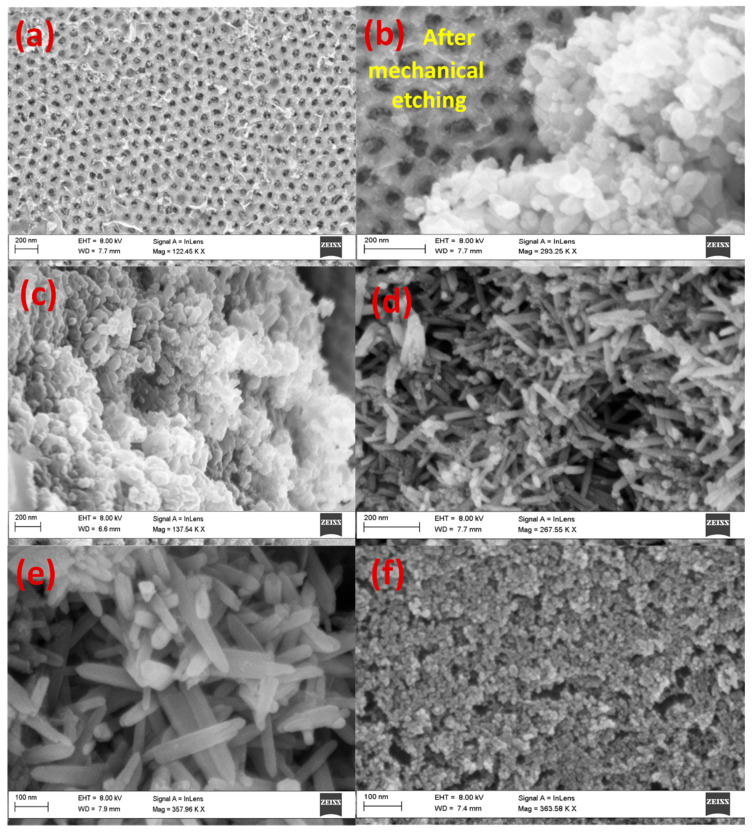
Top-view FE-SEM images of ZnO nanostructures deposited on 10 nm –Au-decorated AAO membranes with a bottom barrier layer at V = 3.2 V (**a**) I = 0.013 A, t = 10 min, (**b**) I = 0.013 A, t = 20 min, (**c**) I = 0.050 A and t = 20 min, (**d**) I = 0.050 A, t = 60 min, and (**e**) I = 0.050 A and t = 90 min, and (**f**) the back side of the membrane after 90 min.

**Figure 7:**
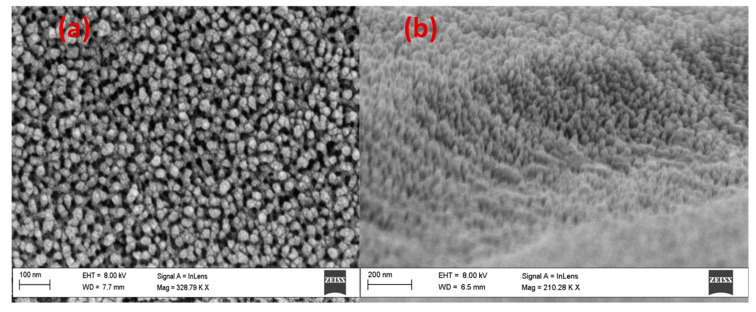
FE-SEM images of blank AAOM electrodeposited with ZnO at I = 0.020 A, V = 3.2 V, and t = 20 min; (**a**) top view and (**b**) oblique view.

To identify the impacts of the bottom barrier layer on the morphology of the generated ZnO nanoarrays, the morphologies of the electrodeposits produced by the direct electrodeposition of ZnO into Au/AAOM and AAOM with barrier layers were investigated and are presented in Figure 6 and Figure 7. Figure 6 shows top-view FE-SEM images of the ZnO nanostructures deposited on 10 nm Au-decorated AAOMs with a bottom barrier layer at V = 3.2 V and (a) I = 0.013 A, t = 10 min, (b) I = 0.013 A, t = 20 min, (c) I = 0.050 A and t = 20 min, (d) I = 0.050 A, t = 60 min, and (e) I = 0.050 A and t = 90 min, and (f) the back side of the membrane after 90 min. This figure shows the different stages for the formation of randomly distributed ZnO nanorods on the top surface of the AAOM. After 10 min deposition at 13 mA, the pores of AAOM were filled to some degrees by the ZnO nanostructures, Figure 6a. When the time was increased to 20 min, the pores were completely blocked, as shown from the etched part in Figure 6b, and ZnO nanoparticles agglomerated on the top surface of AAOM. By increasing the current to 50 mA (Figure 6c), seeds for the growth of ZnO nanorods developed. As the time increased from 20 to 90 min, the sizes of the grown nanorods increased, as shown in Figure 6c–e. Figure 6f shows the surface morphology of the back side of the membrane after 90 min electrodeposition. As seen, the surface of the membrane’s back side is covered with an extremely high density of agglomerated nanoparticles. The sizes of the nanoparticles are less than 10 nm.

Figure 7 shows top-view and oblique-view FE-SEM images of blank AAOM electrodeposited with ZnO at I = 0.020 A, V = 3.2 V, and t = 20 min. This figure shows the formation of high-density ZnO nanoparticles on the top surface of AAOM without obstructing the nanopores.

### 3.4. MB and MO Dye Removal using ZnO/ZnAl_2_O_4_/Au

According to the decrease in methylene blue (MB)- and methyl orange (MO)-dye solution absorbance at their maximal absorption wavelengths, 668 nm and 460 nm, respectively, the dye removal percentage was determined as follows:

(3) $$Dye\;Removal\%=\frac{C_{o}-C_{t}}{C_{o}}\times 100$$

where *C_o_* is the dye solution’s beginning concentration and *C_t_* is the dye solution’s concentration after the time (*t*). For the 50 mL MB and MO solutions of concentrations 20 ppm and using a 3 cm^2^ ZnO/ZnAl_2_O_4_/Au sample, respectively, the effect of the contact time on the dye removal % was conducted for up to 55 min and 180, respectively.

**Figure 8:**
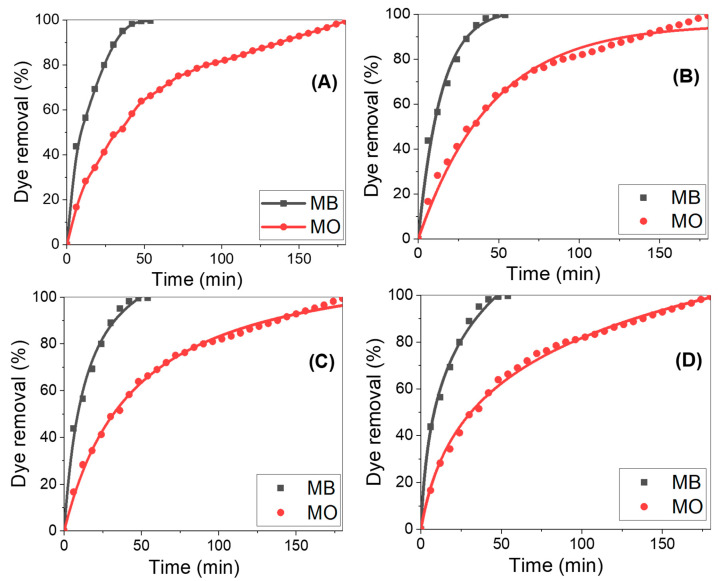
(**A**) MB and MO removal % versus time at a dye concentration of 20 ppm using the ZnO/ZnAl_2_O_4_/Au template, (**B**) nonlinear Pseudo-first-order, (**C**) nonlinear Pseudo-second-order, and (**D**) nonlinear Elovich sorption kinetics of MB and MO dyes at 25 °C and pH 7.

How much dye is removed by adsorption depends significantly on the initial concentration and kind of adsorbate. Figure 8A shows the variations in the amount of MB and MO removed over time, utilizing a nano-adsorbent surface of ZnO/ZnAl_2_O_4_/Au to achieve 100% dye removal. Typically, the dye removal percentage (adsorption) started the process at a rather high level, then steadily dropped until it reached equilibrium. When using new sorbents, the contact time has little effect on the adsorption process until equilibrium has been attained. Because there are so many exposed active adsorption sites on the surfaces of the adsorbent, and because AAOM is being used, the surface-to-volume ratio is huge, which accounts for the quick removal rate during the early phases of adsorption progression. The hot spots were converted into completely occupied MB or MO sites by prolonging the contact duration between the adsorbent and adsorbate. There is an increase in the repulsive forces between dye molecules in the bulk liquid phase and dye molecules adsorbed on adsorbent surfaces as a result [42]. In the case of MB, the rate of adsorption is greater than that of MO. The high driving force for mass transfer at a high starting dye concentration, on the other hand, is the primary factor causing an increase in the amount of dye absorbed by the adsorbent. An adequate increase in the draft forces, therefore, occurs to overcome the mass-transfer barrier between the dye adsorbate and the nano-adsorbent active sites [45,46]. Approximatively, the total elimination percentage for MB and MO dyes at pH 7 and 25 °C is attained after ~50 and 180 min, respectively. The results showed that the used sample yielded higher values than those previously noted for metal-oxide-based adsorbents [47,48,49,50], demonstrating the effectiveness of the used technique in enhancing the dye removal performance.

The three steps of the traditional adsorption mechanism are adsorbate diffusion on the adsorbent’s surface, adsorbate migration in the adsorbent’s pores, and adsorbate layer creation on the adsorbent. The adsorption performance of the sample for MB and MO was evaluated to choose the optimal adsorption kinetics model. Nonlinear graphs of the first-order, second-order, and Elovich kinetics are shown in Figure 8B–D, plotting the percentage of dye removal vs. the time [51,52]. The adsorption kinetics parameters for the assessment model—k_1_, k_2_, q_e_, β, and α, as well as R^2^—were derived using nonlinear fitting and are displayed in Table 2. According to the nonlinear fit and regression coefficient values in Table 2 for all the tested kinetic models, the second-order and Elovich models are efficient at managing MB and MO adsorption onto the suggested nanoadsorbent surface. The close approximation between the calculated dye removal percentage and the experimental removal % served as additional evidence for this. The rate constant for MB was almost triple that of MO, according to the second-order model. The findings of the kinetic analysis presumed that chemisorption was the rate-limiting phase and that the adsorption rate was determined by the adsorption capacity rather than adsorbate concentration.

**Figure 9:**
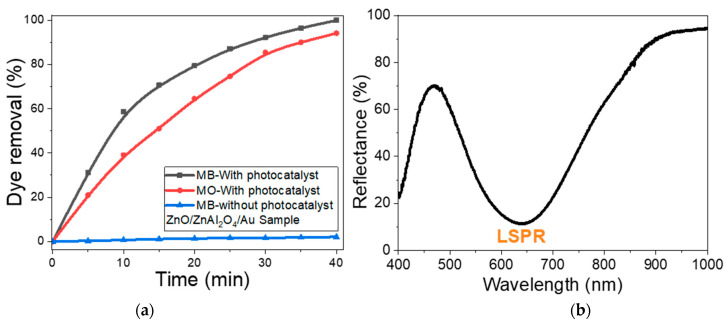
(**a**) The photocatalytic performance of the ZnO/ZnAl_2_O_4_/Au photoelectrode toward the photodegradation % of 20 ppm MB and MO versus time at room temperature and pH 7. For the control test, the MB dye removal % versus light illumination time was presented without the existence of the photoelectrode. (**b**) The reflectance spectrum of ZnO/ZnAl_2_O_4_/Au photoelectrode.

The results of the application of the ZnO/ZnAl_2_O_4_/Au photoelectrode for the photodegradation of 20 ppm MB and MO under white light illumination are shown in Figure 9a. For the control test, only 2.1% removal of MB was reached after 40 min illumination without the existence of the photocatalytic electrode, whereas the existence of the ZnO/ZnAl_2_O_4_/Au photoelectrode increased the removal % to reach 99.9% for MB and 94.1% for MO. These results refer to the high photocatalytic performance of the ZnO/ZnAl_2_O_4_/Au electrode. This may be ascribed to the known semiconducting properties of ZnO [38], the huge surface area of the nanorod array, and the localized surface plasmon resonance (LSPR) of Au nanoparticles [26,39]. The ZnO/ZnAl_2_O_4_/Au photoelectrode’s UV/Vis reflectance spectrum is displayed in Figure 9b. This graph clearly shows the presence of a strong localized plasmonic band centered at 636 nm. This band was closer to the absorption wavelength of MB than MO, which may account for the higher photocatalytic efficacy of the ZnO/ZnAl_2_O_4_/Au photoelectrode toward MB than MO. The plasmonic Au NPs contribute strongly to the efficient photocatalytic process through three main tasks: photon scattering; hot-electron allocation and near-field enhancement [53]. The LSPR may be decayed through the re-emission of photons or the release of active charge carriers. Also, Au NPs may cause multiple reflections of the light to elongate the mean path of the photons in the ZnO/ZnAl_2_O_4_/Au nanoarray [26,53]. The excited electrons on the ZnO/ZnAl_2_O_4_ surfaces are transferred to the surface of Au NPs, which act as current collectors and contribute to the reduction in the charge recombination rate.

### 3.5. pH-Sensing Performance of AAOM/Au and ZnO/ZnAl_2_O_4_/Au Electrodes

**Figure 10:**
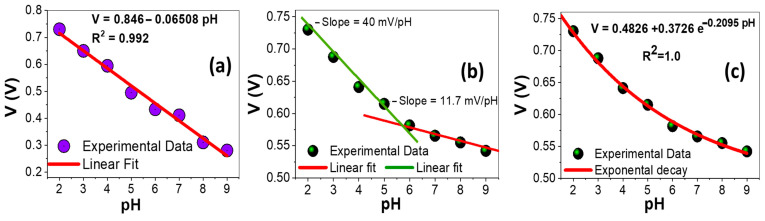
Calibration curve at 25 °C of (**a**) AAOM/Au sensor electrode with linear fitting and (**b**,**c**) AAOM/Au after ZnO deposition (ZnO/ZnAl_2_O_4_/Au sensor electrode), with linear and exponential decay fitting.

Different buffer solutions with a pH range between 2 and 9 at 25 °C were used to study the calibration curves of the AAOM/Au sensor electrode before and after ZnO deposition. When changing the pH value of the buffered solution, the potential dramatically changed, and then spontaneously reached a steady state. The values of potential were collected from steady-state values and presented versus pH values, as shown in Figure 10a,b. These plots provide a standard calibration line for pH sensors.

The sensor’s calibration curves were based on the Nernst Equation (4), where *RT*/*F* = 0.05916, *E°* is the standard electrode potential at 25 °C, *T* is the temperature in kelvin, *R* is a universal gas constant, and *F* is the Faraday constant [54,55,56].

(4) $$E=E^{o}-2.303\;\frac{RT}{nF}\;\mathrm{pH}=E^{o}-0.05916\;\mathrm{pH}$$

The sensitivity of the potentiometric pH sensor can be obtained from the slope of the linear regression according to Equation (4) [57,58]. Based on Nernstian behavior, the theoretical maximum sensitivity was −59.16 mV/pH at 25 °C. The linear fitting resulted in a slope of 65.1 ± 3.2 mV/pH (R^2^ = 0.992) in a wide pH range of 2–9. The sensitivity value of this AAOM/Au was 65.1 mV/pH and the detection limit was pH 12.9. The value of the detection limit was obtained by extrapolating the data line until the intercept with the pH-axis [57,58].

For the ZnO/ZnAl_2_O_4_/Au sensor electrode with nanorods of 98 nm in diameter and 500 nm in length (Figure 10b), two linear segments were found and their linear fitting resulted in a slope of 40.1 ± 1.6 mV/pH (R^2^ = 0.996) in a pH range 2–6. For pH > 6, the slope was reduced to 11.7 ± 0.7 mV/pH (R^2^ = 0.996). This value was very far from the theoretical Nernstian slope. So, we believe that the behavior of this electrode may be non-Nernstian. We used different decay functions to obtain the best fitting of the experimental data. The behavior demonstrated a remarkable exponential decay response to proton H^+^(aq) concentrations in the solution. This sample exhibited a pH-exponential-dependent electrochemical potential difference versus the reference microelectrode. The potential difference was exponentially decayed over a large dynamic range (pH 2–9), as shown in Figure 10c. The sensitivity equation for this electrode was given by V(mV) = 482.6 + 372.6 e^−0.2095 pH^ at 25 °C with R^2^ = 1.0, which could be understood in terms of changes in the surface charge during protonation and deprotonation. Vertically grown nanoelectrodes of this type can be smoothly and gently applied to penetrate a single living cell without causing cell apoptosis. Therefore, these arrays could be developed to create highly sensitive pH sensors for monitoring in vivo biological processes within single cells, which will be the focus of future studies.

Because it is important to test the performance of the sensor with standard commercial solutions and to compare the results with the standard values, the ZnO/ZnAl_2_O_4_/Au sensor electrode was applied to detect three standard HANNA buffer solutions with pH 4.01, 7.01, and 10.01. The data are presented in Table S1 (supplementary data). The measured values of V(mV), the average of triplicate measurements, were applied to obtain pH values from the sensitivity equation of 3.99, 7.03, and 9.84. The error was less than 2%, which indicates good accuracy of the designed pH electrode.

## 4. Conclusions

Zn nanostructure arrays electrodeposited in the nanochannels of the AAOM were oxidized at 300 °C to produce ordered 2D ZnO nanotubes and ZnO/ZnAl_2_O_4_/Au nanorods. According to the Kirkendall effect, the formation of ZnO/ZnAl_2_O_4_/Au nanoarrays was attributed to the complete removal of the AAOM/Au barrier layer, proper relativistic deposition, and the diffusion of Zn^+2^ ions. FE-SEM was used to examine how the electrodeposition current, time, barrier layer, and Au coating affected the morphology of the resulting nanostructures. The structural characteristics and elemental composition study of the ZnO/ZnAl_2_O_4_/Au nanoarray confirmed the Kirkendall effect. The crystallite size of ZnAl_2_O_4_ was 62.28 nm along the preferred orientation (400). The designed ZnO/ZnAl_2_O_4_/Au electrode was applied successfully for the adsorption of MB and MO dyes from aqueous solutions. Using a 3 cm^2^ ZnO/ZnAl_2_O_4_/Au sample, 100% dye removal was achieved after 50 and 180 min, respectively, for 20 ppm MB and MO dyes at pH 7 and 25 °C. From the kinetic study, the adsorption behavior followed the pseudo-second-order model. Almost 100% removal of MB was reached by the ZnO/ZnAl_2_O_4_/Au photocatalytic reaction within 40 min. AAOM/Au and ZnO/ZnAl_2_O_4_/Au nanoarrays were also applied as pH sensor electrodes. The AAOM/Au sensor electrode showed Nernstian behavior, with a sensitivity of 65.1 mV/pH (R^2^ = 0.99) throughout a wide pH range of 2–9 and a detection limit of pH 12.6, whereas the ZnO/ZnAl_2_O_4_/Au electrode showed a sensitivity of 40.1 ± 1.6 mV/pH (R^2^ = 0.996) in a pH range of 2–6. The behavior of the electrode was also fitted with non-Nernstian behavior over the full pH range under examination. Its sensitivity was presented by the exponential decay equation with R^2^ = 1.0. The goal of the future study is to develop a very sensitive pH sensor for monitoring in vivo biological processes in certain cell types using the studied arrays and to study the optical characteristics and photocatalytic performance of different Au coating for more efficient dye removal from wastewater.

## Figures and Tables

**Table 1:** Values of FWHM, relative intensity, crystallite size (CS), d-spacing, and dislocation density.

Pos. [°2Th.]	FWHM [°2Th.]	Rel. Int. [%]	d-Spacing [Å]	Cs [nm]	Dislocation Density [nm^−2^]
38.63	0.315	5.56	2.331	27.9	1.28 × 10^−3^
44.69	0.144	100.00	2.026	62.3	2.58 × 10^−4^
44.81	0.096	52.86	2.026	93.5	1.14 × 10^−4^
78.22	0.144	4.76	1.221	74.2	1.81 × 10^−4^

**Table 2:** Parameters of the nonlinear kinetic models for MB and MO dye adsorption on the surface of the sample.

Pseudo-first order: *Y* = *q_e_* [1 − *exp*(−*k*_1_*X*)]		
MB	$\mathrm{Rate}\;\mathrm{constant}=k_{1}$ (min^−1^)	0.070 ± 0.006
	maximum amount of CR uptake = q_e_ (mg/g)	102.018 ± 2.737
	R^2^	0.9883
MO	*k*_1_	0.022 ± 8.559 × 10^−4^
	q_e_	95.627 ± 1.167
	R^2^	0.9879
Pseudo-second order: $Y=\frac{k_{2}q_{e}^{2}X}{1\;+\;k_{2}q_{e}\;X}$		
MB	$\mathrm{Rate}\;\mathrm{constant}=k_{2}$ (min^−1^)	5.717 × 10^−4^ ± 7.772 × 10^−5^
	q_e_ (mg/g)	127.695 ± 4.254
	R^2^	0.9933
MO	*k*_2_	1.847 × 10^−4^ ± 7.450 × 10^−6^
	q_e_	120.507 ± 1.203
	R^2^	0.9970
$\mathrm{Elovich}\;\mathrm{Kinetic}\;\mathrm{model}:\;Y=\frac{1}{\beta }\ln(\alpha \beta X+1)$		
MB	Adsorption rate at 0 min = α (mg/min)	14.649 ± 1.951
	The extent of surface coverage = β (g/mg)	0.031 ± 0.002
	Correlation Coefficient = R^2^	0.9933
MO	A	3.974 ± 0.152
	Β	0.032 ± 6.770 × 10^−4^
	R^2^	0.99676

## Data Availability

Not applicable.

## Supplementary Materials

The following supporting information can be downloaded at: https://www.mdpi.com/article/10.3390/nano13192667/s1, Figure S1: Point EDX spectra of ZnO/Au/Al_2_O_4_ nanorod array; (a) Al_2_O_3_/Au, (b) ZnO/Au/Al_2_O_4_, and (c) Al_2_O_3_; Table S1: Results of application of ZnO/ZnAl_2_O_4_/Au electrode for three standard commercial buffer solutions. The values of V(mV) are the average of triplicate measurements. The calibration equation, V(mV) = 482.6 + 372.6 e^−0.2095 pH^, was used to calculate pH values.
