# Supplementary material for: Fabrication of ZnO/ZnAl2O4/Au Nanoarrays through DC Electrodeposition Utilizing Nanoporous Anodic Alumina Membranes for Environmental Application
Source: Nanomaterials (Basel). 2023 Sep 28;13(19):2667. doi: 10.3390/nano13192667 (PMC10574107; doi:10.3390/nano13192667)
Supplement: Supplementary file 1 [file nanomaterials-13-02667-s001.zip › nanomaterials-2623412-supplementary.pdf]

Supplementary data

Fabrication of $ZnO/ZnAl_2O_4/Au$ Nanoarrays through DC Electrodeposition Utilizing Nanoporous Anodic Alumina Membranes for Environmental Application

Mohamed Shaban

Department of Physics, Faculty of Science, Islamic University of Madinah, Madinah 42351, Saudi Arabia; mssfadel@aucegypt.edu

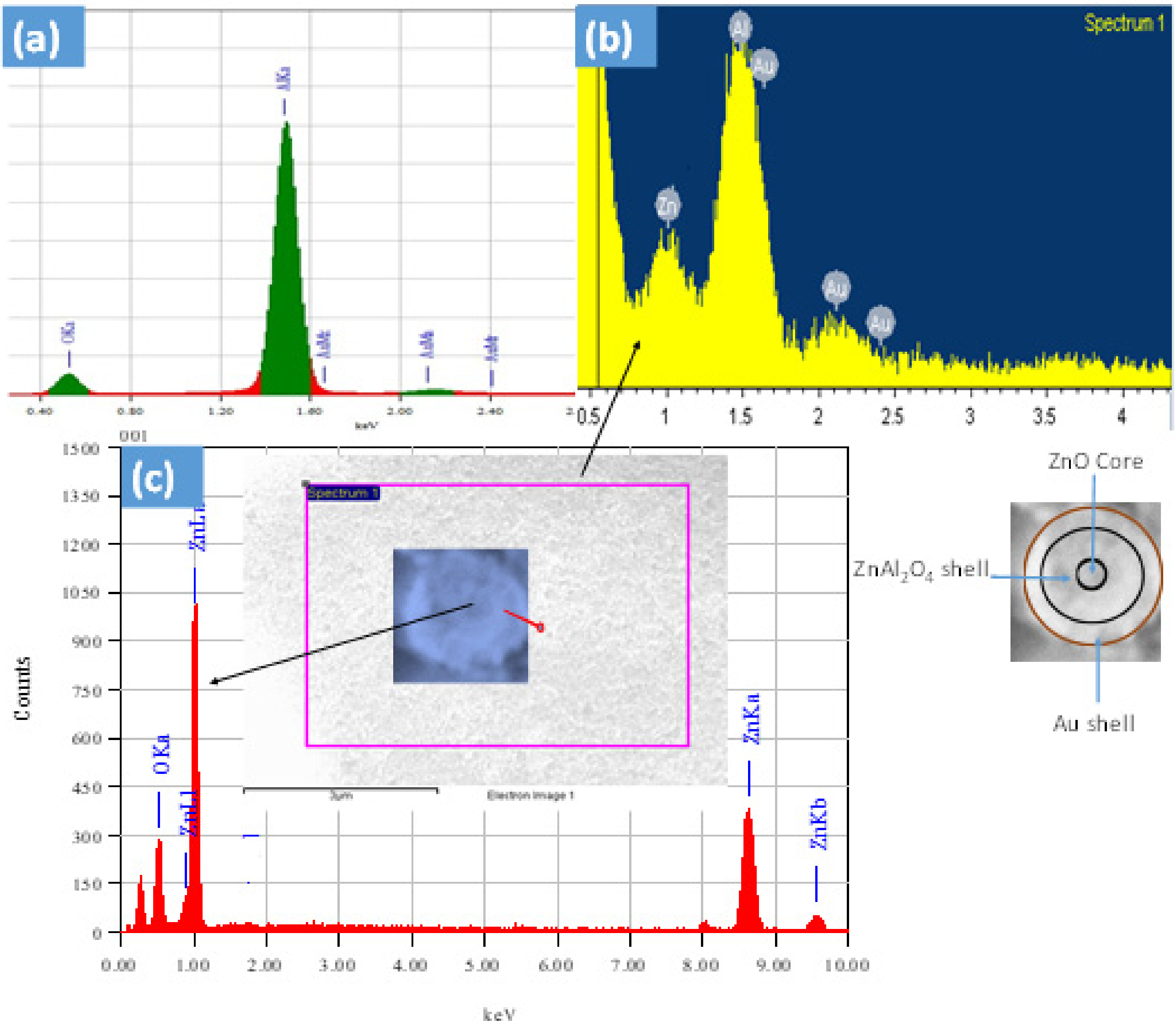

Figure S1. Point EDX spectra of $ZnO/Au/Al_2O_4$ nanorods array; (a) Al_2O_3/Au, (b) $ZnO/Au/Al_2O_4$, and (c) Al_2O_3.

Table S1. Results of application of $ZnO/ZnAl_2O_4/Au$ electrode for three standard commercial buffer solutions. The values of V(mV) are the average of triplicate measurements. The calibration equation, $V(mV) = 482.6 + 372.6\ e^{-0.2095\ pH}$, is used to calculate pH values.

Standard pH	V(mV)	pH value	Error%
4.01	644	3.99	0.41%
7.01	568	7.03	0.31%
10.01	530	9.84	1.68%

.
